# CDK9 as a Valuable Target in Cancer: From Natural Compounds Inhibitors to Current Treatment in Pediatric Soft Tissue Sarcomas

**DOI:** 10.3389/fphar.2020.01230

**Published:** 2020-08-13

**Authors:** Matteo Cassandri, Rossella Fioravanti, Silvia Pomella, Sergio Valente, Dante Rotili, Giada Del Baldo, Biagio De Angelis, Rossella Rota, Antonello Mai

**Affiliations:** ^1^ Department of Oncohematology, Bambino Gesù Children’s Hospital, IRCCS, Rome, Italy; ^2^ Department of Drug Chemistry and Technologies, Sapienza University of Rome, Rome, Italy

**Keywords:** natural inhibitors, CDK9, pediatric sarcoma, gene transcription, chromosomal translocation

## Abstract

Cyclin-Dependent Kinases (CDKs) are well-known reliable targets for cancer treatment being often deregulated. Among them, since the transcription-associated CDK9 represents the sentry of cell transcriptional homeostasis, it can be a valuable target for managing cancers in which the transcriptional machinery is dysregulated by tumor-driver oncogenes. Here we give an overview of some natural compounds identified as CDK inhibitors with reported activity also against CDK9, that were taken as a model for the development of highly active synthetic anti-CDK9 agents. After, we summarize the data on CDK9 inhibition in a group of rare pediatric solid tumors such as rhabdomyosarcoma, Ewing’s sarcoma, synovial sarcoma and malignant rhabdoid tumors (soft tissue sarcomas), highlighting the more recent results in this field. Finally, we discuss the perspective and challenge of CDK9 modulation in cancer.

## Introduction

The cyclin-dependent kinases (CDKs) family includes two main subgroups of kinases, those that mainly regulate cell cycle progression (including CDK1, CDK2, and CDK4/6) and those that control transcriptional processes (including CDK7, CDK8, CDK9, CDK12, and CDK13) (Roskoski, 2019; Chou et al., 2020). For some of the remaining CDKs the functions are still debated and/or under study.

The CDKs activity depends on specific regulatory cyclins subunits (29 cyclins in humans) with which CDKs form heterodimers (Malumbres, 2014). While levels of cell cycle-associated CDKs do not change during the different phases of cell cycle progression, those of their cyclin partners oscillate in a proteasomal-dependent manner thus timely activating selected CDKs (Malumbres, 2014; Chou et al., 2020).

Conversely, cyclins partnered with transcription-associated CDKs are modulated in a cell cycle-independent manner (Malumbres, 2014; Chou et al., 2020). Since transcription-associated CDKs display conserved domains common to the cell cycle-associated ones, including the kinase domain, it has been suggested that cyclins/CDKs dimers can be evolutionary originated in more complex systems to strictly control more specialized cell functions.

Overall, abnormalities in the expression and/or activation of a number of CDKs are considered a hallmark of cancer development and progression (Chou et al., 2020). Over the last years, the importance of CDKs inhibition in cancer has been growing as highlighted by the number of ongoing clinical trials with CDKs inhibitors as single agents and in combination with other drugs (www.clinicaltrials.gov). Most of the CDKs inhibitors developed in the pre-clinical and clinical settings are against the cell cycle-associated subgroup that controls the crucial processes of tumor cell survival and growth. In contrast, the development of small molecules selectively targeting CDKs regulating transcriptional programs has just been started.

Among the transcription-associated CDKs, CDK9 plays a key role in controlling basal gene transcription thus ensuring cell transcriptional homeostasis (Bacon and D’Orso, 2019). This crucial function and the evidence that transcriptional programs are dysregulated in cancer made CDK9 an attractive target for anti-cancer therapies. In agreement, modulation of CDK9 has been recently shown as a promising strategy to hamper the transcriptional machinery in tumors types that are strictly dependent on aberrant transcription from tumor-driver oncogenes (Tibes and Bogenberger, 2019; Chou et al., 2020).

A group of soft tissue sarcomas (STSs) of childhood express chimeric transcription factors from which they are dependent for survival or have mutations or loss of key components of transcriptional complexes (Finetti et al., 2020; Knott et al., 2019). STSs include highly metastatic sub-entities often unresponsive to conventional therapies and characterized by dismal prognosis. Therefore, research on novel approaches aimed at preventing the progression of the disease and, in the meantime, at reducing the morbidity of aggressive therapeutic strategies in young patients is endlessly under way. CDK9 inhibition has been recently investigated in these STSs with naturally- or synthetically-derived compounds.

In this review, we briefly describe the roles of CDK9 in transcriptional processes and summarize the last insights on natural products as CDK9 inhibitors in cancer. We also give an overview of the results of CDK9 inhibition in a subgroup of transcriptionally-driven pediatric STSs. Perspectives and challenge of potential clinical applications of CDK9 inhibitors are also discussed.

## How CDK9 Works

CDK9 consists of two isoforms encoded by the same gene but transcribed from two different promoters (Liu and Herrmann, 2005). The short isoform is the amplest one, mainly distributed in the nucleus and the most involved in transcription (Chao and Price, 2001; Jonkers and Lis, 2015). The long isoform primarily localizes in the nucleolus and does not seem to be involved in transcription (Liu and Herrmann, 2005; Jonkers and Lis 2015).

**Figure 1:**
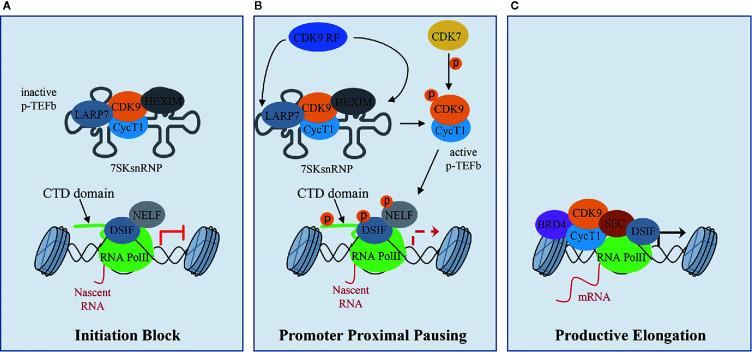
CDK9 function in RNA Pol II activation and gene transcription: **(A)** CDK9 associates with cyclin T1 (CycT1) forming the positive transcription elongation factor b (P-TEFb) complex. The P-TEFb complex is inactive when incorporated into the 7SK small nuclear ribonucleoprotein complex (snRNP) which includes HEXIM. LARP7 interacts with 7SKsnRNP to increase complex stability. RNA polymerase II (RNA PolII) is loaded with the negative elongation factors negative elongation factor (NELF) and DRB sensitivity-inducing factor (DSIF), blocking transcription elongation. **(B)** Upon transcriptional stimuli, CDK9 release factors (CDK9 RF) post-translationally modifies the 7SKsnRNP/P-TEFb complex to promote the release of P-TEFb. To this process concurs the activation/phosphorylation of CDK9 by CDK7, which works as a CDK activating kinase (CAK). Active P-TEFb complex, in turn, phosphorylates DSIF and NELF and the Ser2 of the RNA PolII C terminal domain (CTD) at target promoters to remove elongation blocks. **(C)** Bromodomain protein 4 (BRD4), which is recruited by transcription factors on promoters of target genes that must be activated, recruits, in turn, activated P-TEFb and super elongation complex (SEC) allowing transcriptional elongation and expression of target genes.

CDK9 binds CyclinT1 or CyclinT2 (CycT1, CycT2) with which it forms the core component of Positive Transcription Elongation Factor Complex (P-TEFb), modulating gene transcription by phosphorylating key substrates at serine and threonine residues (Whittaker et al., 2017). Gene transcription is a tightly and timely regulated process beginning with the gene promoter occupancy of the Mediator Complex containing CDK8 or CDK19. The Mediator Complex bridges gene specific activators to RNA Polymerase II (RNA Pol II) transcription machinery to form a firmed pre-initiation complex (Cramer, 2019; Dannappel et al., 2019). Then, RNA Pol II is reversibly phosphorylated at Ser5 and Ser7 by CDK7 present in the Transcription Factor-II H (TFIIH) complex and released from the pre-initiation complex to initiate transcription. After the pre-initiation phase, the RNA Pol II is stuck in pausing state, through the action of the DRB sensitivity-inducing factor (DSIF) and the negative elongation factor (NELF) (**Figure 1A**). During this phase, P-TEFb is maintained in an inactive form through the incorporation into the 7SK small nuclear ribonucleoprotein complex (snRNP) by the binding to CycT1 (**Figure 1A**) (Haaland and Herrmann, 2003; Li et al., 2005). Within this complex, P-TEFb interacts with hexamethylene Bis-acetamide-inducible protein 1/2 (HEXIM1/HEXIM2), which suppresses CDK9 kinase activity (Romano and Giordano, 2008). Finally, lupus antigen related protein 7 (LARP7), binds to 7SK snRNA to increase the complex stability (**Figure 1A**) (Matrone et al., 2015). Subsequently, CDK7 activates CDK9 by phosphorylation, working as a CDK-activating kinase (CAK) (Dannappel et al., 2019) and, in turn, activated CDK9 releases RNA Pol II from pausing state (**Figure 1B**). To be released, and consequently activated, CDK9 also needs the action of the CDK9 release factors (CDK9 RF), which are enzymes that post-translationally modify 7SK snRNP complex subunits (**Figure 1B**) (Chen et al., 2008; Wang et al., 2008). Then, the active P-TEFb CDK9/CycT complex is recruited by bromodomain-containing protein 4 (BRD4) and the super elongation complex (SEC), and phosphorylates three main substrates: Ser2 of RNA Pol II C-terminal domain (CTD), the DSIF and the NELF (**Figure 1B**) (Wada et al., 1998; Yamaguchi et al., 1999; Gomes et al., 2006; Rahl et al., 2010). These events facilitate the release of the RNA Pol II and activate the productive elongation phase of transcription (**Figure 1C**) (Adelman and Lis, 2012; Jonkers and Lis, 2015). Furthermore, CDK9-dependent phosphorylations play a key role in the recruitment of the 3’end processing and splicing factors, responsible for messenger RNA (mRNA) maturation (Yang et al., 2005; Coin and Egly, 2015).

Given its role in basal transcription regulation, CDK9 activity must be strictly regulated and this regulation mainly occurs by protein-protein interactions, as highlighted by the formation of an inactive P-TEFb complex (**Figure 1A**). Another layer of regulation is represented by the CDK9 auto-phosphorylation on T186 in CDK9 T-loop domain, which regulates both CDK9 activity and interaction with CycT and is necessary for the incorporation in the 7SK snRNP complex (Li et al., 2005; Baumli et al., 2008; Larochelle et al., 2012). In turn, CDK9 T-loop dephosphorylation by PP2B and PP1α phosphatases in response to Ca^2+^ signaling promotes the release of CDK9 from the 7SK snRNP complex (Chen et al., 2008; Wang et al., 2008). Moreover, PPM1G, promotes CDK9 T-loop dephosphorylation after NF-kB induction stimulus and, interacting with HEXIM, blocks the re-incorporation of CDK9/CycT in the 7SK snRNP complex (Wang et al., 2008; McNamara et al., 2013). Also, the CycT acetylation by p300 acetyl transferase promotes CDK9/CycT complex dissociation from HEXIM, facilitating the release of CDK9 to induce transcriptional elongation (Cho et al., 2009).

The activity of several co-factors is required for the correct recruitment of CDK9 to a precise genomic region at the right time, in order to properly activate transcription (Barboric et al., 2001; Rahl et al., 2010). NF-kB has been the first discovered transcription factor implicated in CDK9 delivery to transcriptional complexes through the interaction between CDK9 and the RelA subunit (Barboric et al., 2001). Furthermore, CDK9 and MYC, a known oncogenic protein involved in cell cycle progression, have been found to co-occupy promoter regions of MYC gene targets, and the presence of CDK9 is sufficient to drive MYC target genes expression (Galbraith et al., 2019). Moreover, CDK9 participates to *MYC* gene transcription in a number of cancer types through the recruitment of P-TEFb to chromatin by the bromodomain-containing protein 4, BRD4, which recognizes the acetylated histone tails (Lu et al., 2015). Finally, the ability of CDK9 to maintain increased levels of the anti-apoptotic protein MCL1 is considered one of the mechanisms that leads to cancer cell survival and subsequent hematological and solid tumors development (Yin et al., 2014).

## Natural Compounds Acting On CDK9

**Figure 2:**
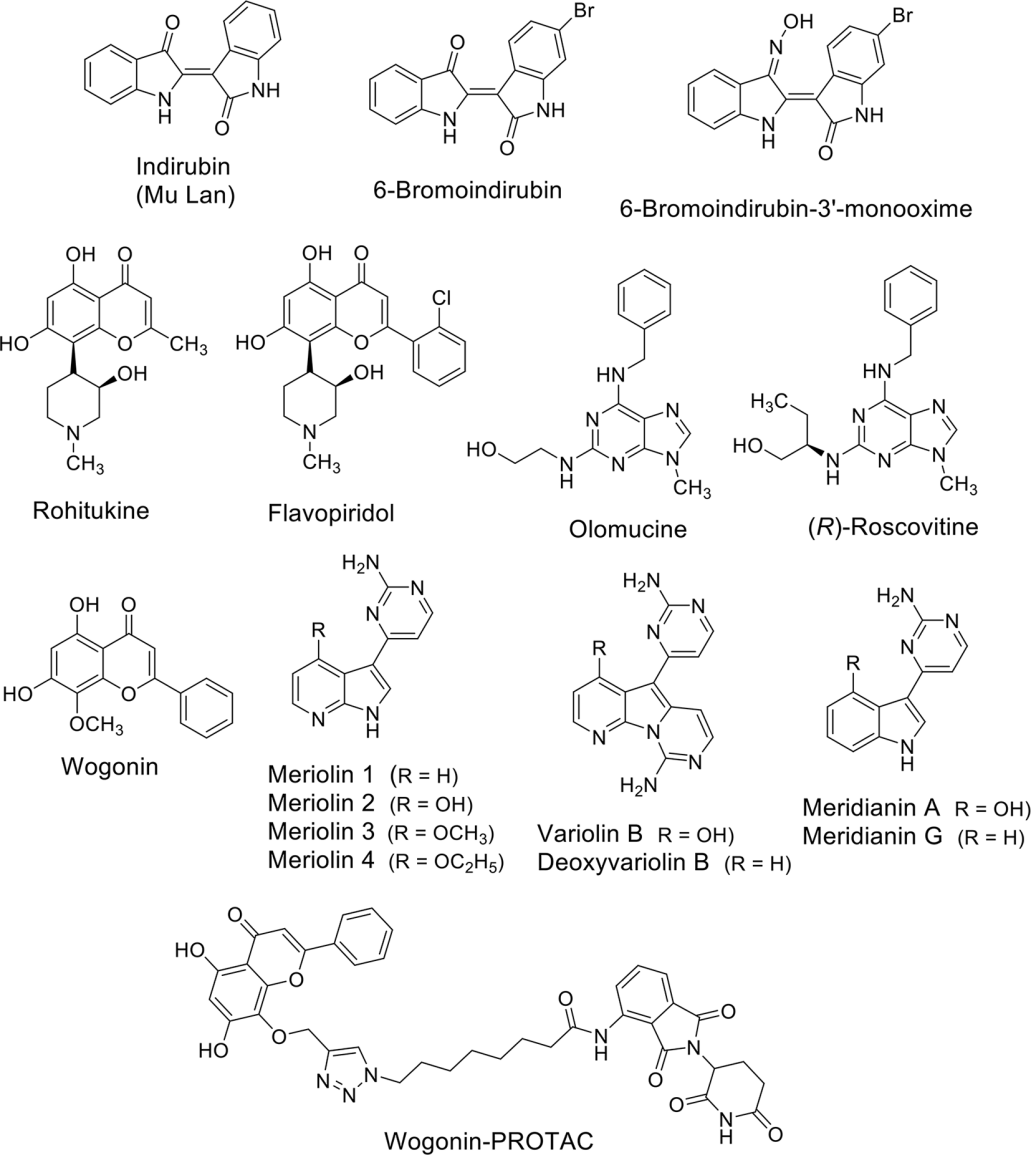
Natural compounds as CDK (CDK9) inhibitors and their synthetic analogues.

A number of natural compounds have been identified as non-selective inhibitors of CDKs. The bis-indoles indirubins were the first human-used compounds to be identified as CDK inhibitors (**Figure 2**). Mu Lan (*Indigofera tinctoria*) has been identified as the major active ingredient of the traditional Chinese medicine formulation known as Danggui Longhui Wan, and has been used for many years to treat chronic myelogenous leukemia (CML) in China (Xiao et al., 2002). Indirubins can be isolated from many indigo-producing plants, by bacteria and by gastropod mollusks, which produce the purplish-red dye known from antiquity as “Tyrian purple.” A series of synthetic substituted indirubins such as 6-bromoindirubin (**Figure 2**) and 6-bromoindirubin-3’-monooxime (**Figure 2**) displayed similar activity as other known CDK inhibitors such as flavopiridol and roscovitine (see below), and are candidates for preclinical development. In particular, 6-bromoindirubin-3’-monooxime has been shown to inhibit CDK9 in addition to CDK2 (Yan et al., 2015).

Rohitukine (**Figure 2**), a constituent isolated from *Dysoxylum binectariferum* and responsible for anti-inflammatory and immunomodulatory activity, was the model for the semisynthetic flavonoid alkaloid flavopiridol (alvocidib) (**Figure 2**). This small molecule has been discovered through a NCI-based screening of about 72,000 compounds on a panel of 60 human cancer cell lines (Weinstein et al., 1997). Its marked effects, antiproliferative and cytotoxic *in vitro* and growth inhibitory *in vivo* in tumor xenografts have been initially ascribed to its ability to inhibit CDK2, CDK1, and CDK4 and with a lesser extent CDK7 (Kaur et al., 1992; Patel et al., 1998). Later, it has been demonstrated that flavopiridol potently inhibits CDK9 (Chao and Price, 2001).

Unfortunately, despite its remarkable pre-clinical effects, it does not demonstrate significant activity in clinical studies on patients with cancers with the exception for those affected by hematological malignancies and, in particular by chronic lymphocytic leukemia (CLL) (Lin et al., 2010; Chou et al., 2020). One of the problems related to flavopiridol administration is that, as the majority of non-selective CDKs inhibitors, it demonstrated heavy side effects for patients. An interesting study specifically inhibiting CDK9 using a dominant-negative form of the protein showed that the number of transcriptionally-inhibited genes is markedly lower than that obtained after flavopiridol treatment (Garriga et al., 2010). This suggests that the compound lacks of selectivity and could also explain the adverse effects on patients anticipated by the evidence that it is partially cytotoxic also on normal cells (Liu et al., 2012).

Olomucine (**Figure 2**), originally isolated from the cotyledons of radish (*Raphanus sativus*), is a CDK inhibitor (Whittaker et al., 2007) that furnished the natural product model for the synthetic drug roscovitine (Seliciclib), the (*R*) enantiomer of a 2,6,9-trisubstituted purine (**Figure 2**) (Meijer and Raymond, 2003). Roscovitine is a CDK2/7/9 inhibitor in clinical trials for the treatment of non-small cell lung cancer (NSCLC), Cushing’s disease, leukemia, multiple myeloma, HIV infection, herpes simplex infection, cystic fibrosis, and chronic inflammation disorders. In addition to CDK inhibition, roscovitine is also able to inhibit RNA polymerase II-dependent transcription and to induce down-regulation of the protein MCL1 (MacCallum et al., 2005). In NSCLC and other cancers roscovitine induces apoptosis, while the side-effects reported in phase I trials are nausea, vomiting, transient elevations in serum creatinine and liver function parameters and transient hypokalemia.

Wogonin (**Figure 2**) is a natural flavone extracted firstly in China from the medicinal plant *Scutellaria baicalensis*. Wogonin exhibits antinflammatory and antiallergic properties and, more recently, has shown anti-cancer efficacy and has been tested in clinical studies (www.clinicaltrials.org). The main anti-cancer mechanism of wogonin and other related flavones such as apigenin, chrysin, and luteolin, has been ascribed to their ability to selectively inhibit CDK9 activity by binding the ATP-binding pocket (Polier et al., 2011). In leukemic cells, the inhibition of the phosphorylation of the CTD of RNA Pol II at the Ser2 sites after treatment with these compounds was associated to the down-regulation of the anti-apoptotic protein MCL1 and followed by apoptosis (Polier et al., 2011). The same effect was obtained in different cancer cell lines such as Hodgkin’s lymphoma, melanoma, hepatocellular, and pancreatic carcinoma, and breast cancer cells suggesting modulation of common pathways. Importantly, wogonin showed preferential killing effects on tumor cells compared to normal cells. Moreover, wogonin and related flavones increased the effectiveness of the BCL2-family inhibitor ABT-263 in combinatorial treatments not only in ABT-263 sensitive but also in ABT-263 resistant cell lines and in primary leukemia cells (Polier et al., 2015).

The group of Meijer (Meijer and Raymond, 2003) reported the synthesis of a family of inhibitors, meriolins (**Figure 2**), with a hybrid structure derived from the two different natural compounds from marine organisms, variolins, and meridianins (**Figure 2**), which display chemical similarity (Bettayeb et al., 2007). Variolin (VAR-B) was isolated from the sponge *Kirkpatrickia variolosa* two decades ago (Simone et al., 2005) and, in particular its more stable deoxy-derivative (dVAR-B) was used on multiple cancer cell lines showing pro-apoptotic properties and anti-tumorigenic effects *in vitro* (Simone et al., 2005). Meridianins were isolated from the Ascidian *Aplidium meridianum* (Franco et al., 1998) but showed limited antiproliferative effects. Unlike the compounds from which they derived, meriolins exerted anti-cancer effects *in vivo* and showed pro-apoptotic activity *in vitro* selectively inhibiting CDKs, including the CDK9-dependent phosphorylation RNA Pol II on Ser2, thus leading to consequent down-regulation of MCL1 and tumor cell death (Bettayeb et al., 2007).

Interestingly, the group of Li used wogonin as scaffold to design and synthesize, through a “click chemistry” approach, proteolysis targeting chimeras (PROTACs) targeting CDK9 by the recruitment of the ubiquitin E3 ligase cereblon (CRBN) (**Figure 2**) (Bian et al., 2018). They identified one of the compounds able to degrade CDK9 through a proteasome- and CRBN-dependent mechanism, moderately inhibiting breast cancer cells proliferation possibly *via* MCL1 down-regulation. Overall, even if natural products can have low selectivity against specific CDKs isoforms, they can also be used as scaffolds to develop more selective degraders in the future.

## CDK9 Blockade as A Potential Treatment For Pediatric Soft Tissue Sarcomas

Pediatric soft tissue sarcomas (STSs) are rare malignancies of children and adolescents that accounts for 8% of all pediatric cancers (Kattner et al., 2019). They are highly heterogeneous under a cellular and genomic point of view including subgroups mainly characterized by chromosomal translocations or genomic abnormalities (Gröbner et al., 2018; Knott et al., 2019). Indeed, STSs have a low number of gene mutations, as pediatric cancers in general (Monje, 2018), and some of them do not have any mutation, suggesting that developmental epigenetic dysregulations rather than genetic alterations could be involved in their pathogenesis (Shern et al., 2014; Tirode et al., 2014; Pishas and Lessnick, 2016).

In agreement with this hypothesis, investigations on chromatin remodelers and transcription factors (TFs) as well as transcription-associated factors have yielded important results in preclinical research on STSs in the last years. In particular, STSs whose cells are dependent from fusion proteins (translocations positive) working as oncogenic TFs or co-activator of oncogenic transcription, such as rhabdomyosarcoma (RMS), Ewing’s sarcoma (ES), and synovial sarcoma (SS), and or from mutated components of transcriptional complexes such as malignant rhabdoid tumors (MRT), have shown promising response to modulations of the gene transcription machinery. Core regulatory complexes of oncogenic TFs drive transcriptional programs in these tumors by binding to enhancer regions (Gollavilli et al., 2018; McBride et al., 2018; Gryder et al., 2019a; Gryder et al., 2019b). Enhancers are short non-coding DNA regions located distally from a promoter region, whose function “enhances/potentiates” the levels of expression of a *cis*-encoded gene (Spitz and Furlong, 2012). In this view, being core regulatory TFs considered undruggable or very difficult to target, inhibition of transcription-associated enzymes such as CDK9 can represent an approach to indirectly target the oncogenic transcriptional machinery.

### CDK9 Inhibition in Rhabdomyosarcoma

PAX3-FOXO1 is a chimeric oncogenic TF expressed by high risk RMSs characterized by t(2;13) chromosomal translocation (~ 20% of cases) (Missiaglia et al., 2012). Fusion-negative RMSs present a low number of recurrent genomic abnormalities and often mutations of the RAS pathway (Shern et al., 2014; Skapek et al., 2019). RMS originates from mesenchymal precursors but, although RMS cells express the skeletal muscle-specific master TFs MYOD and MYOG, they are unable to undergo terminal muscle differentiation, i.e., myogenesis (Taulli et al., 2009; Raimondi et al., 2012). Several causes have been hypothesized and partly demonstrated to explain this malignant feature including alterations in CDK9 activity.

Conversely to the cell cycle-associated CDKs such as CDK1 and CDK2, CDK9 and CycT2 are not down-regulated during myogenesis (Simone et al., 2002). It has been reported that, at the onset of differentiation, MYOD recruits *in vitro* the p300 and PCAF acetyltransferases and the SWI/SNF ATP-dependent chromatin remodeling complex, which includes BAF complex families, together with CDK9/CycT2 on promoters/enhancers regions to activate high level transcription of muscle-specific genes (Giacinti et al., 2006). This seems to be in agreement with the evidence that the CDK9/CycT2 complex can directly interact with the DNA-binding domain of MYOD (the bHLH region) *in vitro* and phosphorylate MYOD on Ser37, to potentiate MYOD-dependent transactivation (Simone et al., 2002; Simone and Giordano, 2007).

In this scenario, p38αβ MAPK kinase (hereafter p38) regulates myoblasts-to-myotubes transition fostering the activity of MYOD also by promoting CycT2 binding to DNA with consequent CDK9/CycT2 complex activation (Simone and Giordano, 2007). To do so, p38 needs an unaltered CDK9/CycT2 complex to exert its promyogenic functions. Simone and Giordano (2007) showed that in a RAS mutated RMS cell line, although the level of CDK9/CycT2 was similar if not higher to that in myoblasts, the enzymatic complex was unable to phosphorylate MYOD even when transiently over-expressed. Being p38αβ MAPK kinase activity defective in RMS cells (Puri et al., 2000), and its activity necessary to promote CDK9 recruitment on DNA of muscle genes by MYOD (Simone and Giordano, 2007), it is conceivable that CDK9/CycT2 complex does not work properly in RMS cells. Interestingly, in contrast to what suggested with previous works, Yu *et al*. reported that the knock down (KD) of CDK9 facilitates rather than inhibits the MyoD-dependent transcription of *MYOG* and muscle genes in differentiating murine myoblasts (Yu et al., 2019). In this cellular model of myogenesis *in vitro*, no CDK9-MYOD interaction was detected, and CDK9 dissociated from CycT1 leading to P-TEFb and BRD4-P-TEFb complex levels decrease. Moreover, in proliferating murine myoblasts CDK9 was found associated to the gene locus of the proliferating gene *MKI67* (also known as *MIB-1*) and its KD restrained cell number expansion (Yu et al., 2019). In agreement with these last data, CDK9 inhibition in multiple cancer cells leads to the de-repression of tumor suppressor genes due to dephosphorylation of BRG1, a component of SWI/SNF complex, and results in cell differentiation (Zhang et al., 2018). Overall, studies are still needed to identify additional potential targets of CDK9 and its partners and to clearly define its role in different cell contexts.

Recently, we demonstrated that PAX3-FOXO1, whose expression is strictly needed for tumor cell survival (Gryder et al., 2017), forms a core regulatory transcriptional complex that involves MYOD, MYOG, MYCN, and BRD4 to activate the oncogenic transcriptional program by binding to clusters of enhancers, i.e., super-enhancers (SE) (Gryder et al., 2019b). Using a luciferase reporter driven by an intronic SE within the gene locus of the PAX3-FOXO1 target gene *ALK*, we showed that while BRD4 inhibitors clearly displayed selectivity for SE-driven luciferase compared to constitutively active promoter-driven luciferase, the CDK9 inhibitor flavopiridol had a general transcriptional inhibitory effect. Further experiments could clarify both the enhancer inhibition selectivity of flavopiridol and the specific role of CDK9 in SE activation in the context of fusion-positive RMS.

### CDK9 Inhibition in Ewing’s Sarcoma

**Figure 3:**
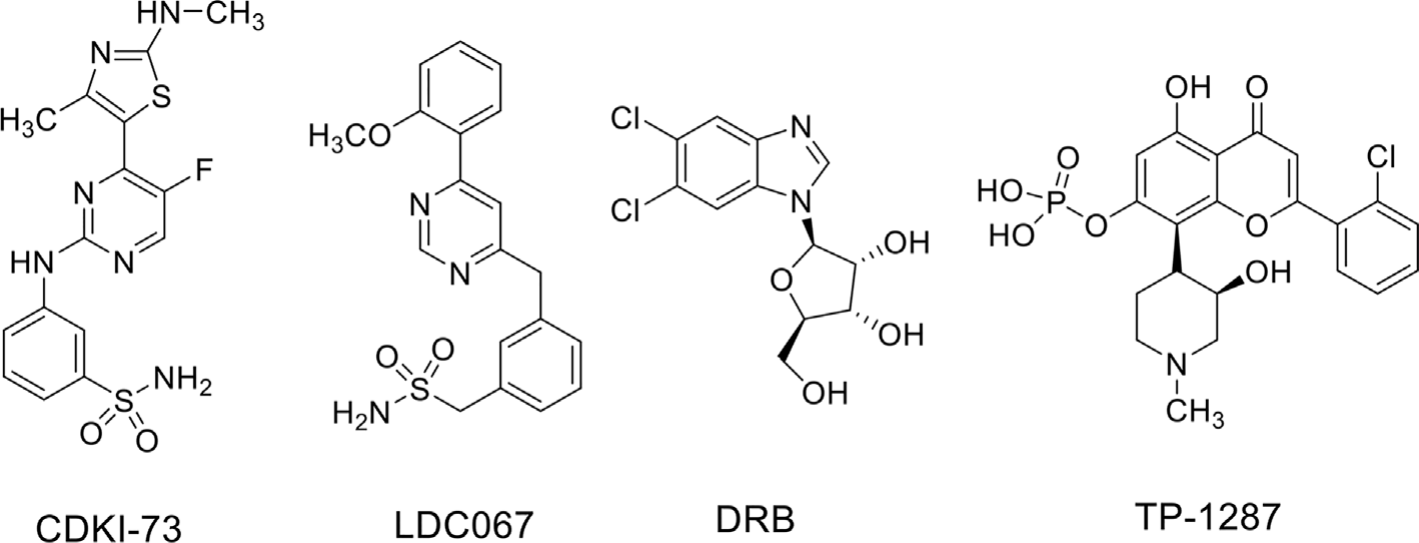
CDK9 inhibitors used in soft tissue sarcomas.

**Figure 4:**
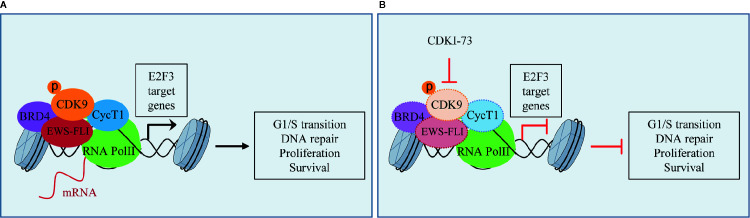
CDK9 function in Ewing’s sarcoma (ES): **(A)** activated p-CDK9/CycT1 complex (known as P-TEFb) binds to bromodomain protein 4 (BRD4) and the fusion protein EWS-FLI to induce the transcription of E2F3 target genes and promotes G1/S cell cycle transition, DNA repair, proliferation, and cancer cell survival. **(B)** CDK9 inhibitor CDKI-73 reduces the expression of CDK9, EWS-FLI, CycT1, and partly that of BRD4, thus inhibiting E2F3 transcriptional program and the tumorigenic features.

ES is a rare pediatric STS that can also arise in bone and has highly invasive properties, with less than 20% of overall survival rates when metastatic (Arnaldez and Helman, 2014). The ES shows chromosomal translocations the most frequent of which is the t(11;22), which involves *EWSR1* and the *FLI1* gene of *ETS* family (Riggi et al., 2014). The resulting chimeric transcription factor EWS-FLI1 can act both as a transcriptional repressor or activator (Riggi et al., 2014). EWS-FLI1 is able to bind to microsatellite chromatin regions and activate SE-dependent transcription of oncogenic target genes (Riggi et al., 2014). EWS/FLI1 requires BRD4 to perform its oncogenic transcriptional program (Hensel et al., 2016), among which to induce the expression of the tumorigenic repressor PHF19 (Gollavilli et al., 2018). Among natural compounds acting as CDK9 inhibitors, the hybrid natural compound meriolin 3 (**Figure 2**) was one of the first tested on ES cell lines *in vivo* showing marked inhibition of tumor growth (Bettayeb et al., 2007). More recently, an interaction among BRD4, p-CDK9, and EWS-FLI1 was demonstrated, whose global expression was reduced by a synthetic CDK9 inhibitor, the 4-(5-thiazolyl)pyrimidine CDKI-73 (**Figures 3** and **4**) (Richter et al., 2020). Indeed, active P-TEFb was able to induce E2F target genes necessary for the G1/S transition, affecting DNA replication and mitotic activity, which was inhibited by CDKI-73, thus confirming a role for CDK9 in proliferation and survival of ES cells (**Figure 4**) (Richter et al., 2020). Interestingly, the inhibition of CDK9 was used by the authors to revert the resistance to JQ1, a BRD4 inhibitor, observed in some ES cell lines, and resulted in more effective induction of apoptosis and tumor growth inhibition *in vivo* than the single agents, indicating that BRD4i and CDK9i combined treatments can be of therapeutic advantage (Richter et al., 2020). Thereafter, CDK9 inhibition has been exploited to reduce the toxicity and increase the effectiveness of a small molecule targeting EWS-FLI1, called mithramycin, which is toxic for liver and thus cannot be delivered at the doses to reach the right blood concentration to block the functions of the fusion protein (Flores et al., 2020). In this case, CDK9 inhibition acted as a potentiator of mithramycin enhancing the suppression of the EWS-FLI1 transcriptional program lowering the IC50 in xenografted ES models. In addition, inhibiting CDK9 strongly reduced the cytotoxicity in the liver cancer-derived HepG2 cells *in vitro* decreasing the expression of *BTG2*, a gene involved in the production of cytotoxic ROS in liver (Flores et al., 2020).

### CDK9 Inhibition in Synovial Sarcoma

SS is the most common non-rhabdomyosarcoma STS in adolescents and young adults (Hale et al., 2019). Conversely to the adult forms, pediatric SS are rarely metastatic at diagnosis (Ferrari et al., 2012a) but has a predisposition to metastasize resulting in 5-years disease free survival or 60% after the first surgery of localized tumor followed by first-line chemotherapy with alkylating agents (Ferrari et al., 2012b; Sultan et al., 2009).

In two third of cases SS is characterized by the chromosomal translocation t(X;18) as the only cytogenetic abnormality, which encodes for the fusion oncoprotein SS18/SSX1 considered the driver of malignancy (Haldar et al., 2007; Naka et al., 2010). Other fusion partners are SSX2 in one third and more rarely SSX4 (as reviewed by Hale et al., 2019). SS18 does not bind DNA but works as a co-activator of transcription by interacting with the SWI/SNF complex to control and drive the expression of a number of gene pathways involved in stemness, cell differentiation, and cell cycle progression (Middeljans et al., 2012). To do so, SS18 can bind in the nucleus to two catalytic ATPase subunits of the BAF complexes, which are mutually exclusive, SMARCA2 and BRG1, to associate with SMARCB1/SNF5/BAF47. Through this process, SS18 concurs to the blockade of HDACs repressor complexes ultimately favoring the transcriptional activation of selected genes (Kadoch and Crabtree, 2013). Conversely, SSX1 is a transcriptional repressor that interacts in the nucleus with BMI1, a polycomb group (PcG) protein of the polycomb repressor complex 1 (PRC1) that, together with the PRC2, promotes chromatin compaction to inactivate gene transcription (Wang et al., 2013). Therefore, the fusion protein can have both functions as an activator and a repressor. The tumorigenic ability of SS18/SSX1 lies on its ability to be incorporated into SWI/SNF complexes evicting the tumor suppressor subunit BAF47 (Kadoch and Crabtree, 2013). Since the functions of BAF complexes are specific for each subunit composition, the BAF47-free SS18-SSX-containing SWI/SNF complexes change their functions and are redirected to PRC2-controlled sites, activating the transcription of genes normally silenced as well as blocking the accessibility to sites previously transcriptionally active (McBride et al., 2018). It appears therefore clear that the pathogenesis of SS is related to deregulation of transcriptional programs.

Recently, the inhibition of CDK9 has been applied to SS cells *in vitro*. Indeed, CDK9 was reported highly expressed in SS cell lines and in samples from patients with high expression associated with poor prognosis (Li et al., 2019). Furthermore, CDK9 genetic KD was able to reduce cell viability and proliferation as well as the levels of Ser2 and Ser5 phosphorylation on RNA Pol II compared to the total protein in two SS18-SSX1-positive SS cell lines. CDK9 pharmacologic inhibition using the inhibitor LDC000067 (LDC067) (**Figure 3**), with nanomolar selectivity against CDK9 over CDK2, CDK6, and CDK7 (Albert et al., 2014), mirrored the anti-tumor effects of CDK9 KD. Moreover, LDC067 induced cell death of SS cells and affected their ability to migrate and to grow in 3D as spheres. These results were associated to the reported down-regulation of MCL1 and survivin levels (anti-apoptotic proteins) and up-regulation of those of the pro-apoptotic protein BAX (Li et al., 2019). Even if the global effect on the transcriptional capacity of the fusion protein has not been verified, this study suggests CDK9 inhibition as a potential approach to hamper the tumorigenic features of SS cells.

### CDK9 Inhibition in Malignant Rhabdoid Tumors

MRT are a subgroup of pediatric cancers arising in soft tissues and characterized by biallelic loss or inactivation of *SMARCB1* or, more rarely, *SMARCA4*, two genes encoding for core components of the SWI/SNF complex (see above and Finetti et al., 2020). MRTs are deadly cancers in which the loss of function of one single component of SWI/SNF complex is pathogenic resulting in aberrant transcriptional function of the entire complex. With the attempt to hinder the deregulated transcriptional machinery in MRT, Moreno et al. (2017) inhibited both BRD4 and CDK9 with small compounds, DRB (**Figure 3**) and LDC067 as CDK9i and JQ1 and I-BET as BRD4i in MRT cell lines *in vitro*. MRT cells showed cell cycle arrest in the G1 phase of the cell cycle and apoptosis with single agents at lower levels for the more specific inhibitors, and the effects were amplified for combinatorial treatments. Moreover, combined treatment appeared synergistic in the slowing down tumor growth *in vivo* (Moreno et al., 2017). Only CDK9 inhibitors as single agents impaired the transcription of the anti-apoptotic genes *MCL1*, *BCL6*, and *BTG1*, which was synergistically decreased with combined treatments. Both CDK9 and BRD4 inhibitors showed modest effects on *MYC* expression while the combination strongly decreased its nascent mRNA and protein levels. Interestingly, the general transcription process was also affected by CDK9 or CDK9/BRD4 combined inhibition as demonstrated by down-regulation of housekeeping genes *RPL3* and *GAPDH*.

## Concluding Remarks

A number of clinical trials have been activated using flavopiridol as single agent or, more often, in combination with other conventional chemotherapeutics in the last decades, the majority of them completed or terminated (**Table 1**). Only one phase I clinical study with flavopiridol has been done exclusively on patients with cancers aged ≤ 21 years of age, and included two patients with RMS and one patient with ES, for which there are results but no following clinical studies (**Table 1**) (Whitlock et al., 2005). Recently, in order to facilitate prolonged repression of the CDK9 target and anti-apoptotic protein MCL1 through chronic dosing and scheduling of flavopiridol, which can be only delivered intravenously because its poor solubility, an oral phosphate prodrug, TP-1287 (**Figure 3**), has been synthesized. This phosphate prodrug has an improved solubility under neutral or basic conditions allowing oral administration of the drug. Pharmacodynamic and efficacy studies in acute myeloid leukemia (Kim et al., 2017) and prostate cancer (Forostyan et al., 2019) xenografted models, showed that orally delivered TP-1287 is efficiently metabolized and enhances tumor regression *in vivo*. A phase I study using oral TP-1287 is actively recruiting to determine the maximum tolerated dose (MTD) and dose-limiting toxicities (DLTs) in patients with advanced metastatic or progressive solid tumors who are refractory to established therapy (**Table 1**).

**Table 1:** Clinical trials of natural products targeting CDK9 used as single agents or in combination in solid tumor including sarcomas.

Agent	Clinical phases	Combination treatment	Disease type	Status	NCT number
**Alvocidib** Flavopiridol	I		* Children with relapsed or refractory solid tumors or lymphomas	Completed	NCT00012181	
	I	Doxorubicin	Gastrointestinal stromal tumor/recurrent and stage IV adult soft tissue sarcoma	Completed	NCT00098579	
	I	Fluorouracil/oxaliplatin/leucovorin calcium	Adult solid tumor	Completed	NCT00080990	
	I		Adult solid tumor	Terminated	NCT00112684	
	I	Vorinostat	Adult solid tumor	Completed	NCT00324480	
	I	Gemcitabine/irinotecan	Adult solid tumor	Completed	NCT00079352	
	II		Sarcoma	Completed	NCT00005974	
	I	Fludeoxyglucose F18/fluorine F18 fluorothymidine	Hematopoietic/lymphoid cancer/adult solid tumor	Terminated	NCT00070239	
	I	Gemcitabine	Adult solid tumor	Completed	NCT00072436	
	I	Irinotecan/cisplatin	Adult solid tumor	Completed	NCT00046917	
	I		Lymphoma/prostate cancer/small intestine cancer/adult solid tumor	Completed	NCT00019344	
	I	Gemcitabine	Adult solid tumor	Completed	NCT00007917	
	I	Leucovorin calcium/fluorouracil/irinotecan	Adult solid tumor	Completed	NCT00021073	
	I	Docetaxel	Adult solid tumor	Completed	NCT00016185	
	I	Irinotecan	Adult solid tumor	Completed	NCT00006485	
	I	Irinotecan/fluorouracil/leucovorin calcium	Adult solid tumor	Completed	NCT00042874	
	I	Docetaxel	Adult solid tumor	Completed	NCT00045448	
	I	Cisplatin/paclitaxel	Adult solid tumor	Completed	NCT00003004	
**TP-1287**	I		Advanced solid tumors	Recruiting	NCT03604783

*Pediatric trial. Source: www.clinicaltrial.gov (March 2020).

One of the key aspects in the evaluation and optimization of transcription-associated CDKs inhibitors is to develop a quantitative, robust, and selective cell-based assay. This step is made difficult by the lack of cellular functions specific to and/or substrates exclusively phosphorylated by each CDK. Li et al. (2019) recently developed a method to check the effects of selective inhibition of CDK8/19. They developed a luciferase-driven assay using a plasmid expressing an NF-kB-dependent consensus promoter, which needs the cooperation of CDK8/19 and NF-kB proteins to be activated. Then, they transfected wild-type packaging kidney HEK 293 cells and, in parallel, matching cells which have been knocked out (KO) for CDK8/19 by a CRISPR/Cas9 system. The CDK8/19 KO cells represented a selectivity control for the dependence on the two enzymes when results of treatment with small molecules was compared to those obtained in wild-type cells (Li et al., 2019). This method could be also used to identify novel effective CDK9 inhibitors.

Moreover, not only the selectivity for a specific kinase but also the secondary effects related to the kinase complete inhibition should be taken into account for treatment on patients. For example, CDK9 phosphorylates p53 and flavopiridol prevents p53 phosphorylation by P-TEFb and by multiple kinases *in vitro* (Radhakrishnan and Gartel, 2006), thus impairing p53 to bind DNA and to activate the transcription of *CDKN1A*/p21, one of the main effectors of p53-dependent cell cycle arrest and apoptosis (Radhakrishnan et al., 2008). Therefore, the use of P-TEFb inhibitors must be considered with caution as they could compromise the effect of DNA-damaging agents that induce tumor cell death *via* p53 activation in p53 wild-type tumors. Moreover, a decreased of p53 levels enhances the transcription of *NPAT* gene locus, which encodes for a p-TEFb-associated protein needed for histone gene expression, and this concurs to the activation of the DNA repair mechanism, a process that if altered promotes the progression of damaged cells through cell cycle (Pirngruber and Johnsen, 2010). Finally, it has been recently reported that CDK9 exerts also a function on heterochromatin maintaining gene silencing thus amplifying the potentiality of this kinase (Zhang et al., 2018). In conclusion, several clinical studies are ongoing using CDK9 inhibitors in association with other compounds, whose results will give information about the feasibility of such approach. Overall, additional future preclinical research is needed to better understand how CDK9 functions in normal and cancer cells to ultimately define the therapeutic applicability of CDK9 inhibitors as single or adjuvant anti-cancer molecules.

## Author Contributions

RR and AM conceived the paper and selected the literature. All the authors participated in the writing and/or correction of the paper. All authors contributed to the article and approved the submitted version.

## Funding

The present work was supported by grants from Associazione Italiana per la Ricerca sul Cancro (AIRC Project # 15312 to RR, AIRC Project # 19162 to AM); Italian Ministry of Health Ricerca Finalizzata (Project # PE-2013-02355271) to RR and AM; Italian Ministry of Health Ricerca Corrente 2020 to RR; PRIN 2016 (prot.20152TE5PK) to AM, and Progetto di Ateneo Sapienza 2017 no. RM11715C7CA6CE53 to DR. SP is a recipient of a Fondazione Veronesi 2020 fellowship.

## Conflict of Interest

The authors declare that the research was conducted in the absence of any commercial or financial relationships that could be construed as a potential conflict of interest.
